# Resistance of E2F4DN to p38^MAPK^ phosphorylation reduces genotoxic cell death in N2a neuron-like cells

**DOI:** 10.1038/s41598-026-54592-z

**Published:** 2026-05-30

**Authors:** Aina M. Llabrés-Mas, Noelia López-Sánchez, Alberto Garrido-García, Vanesa Cano-Daganzo, Irene Camacho-Olmos, José María Frade

**Affiliations:** 1 Department of Molecular, Cellular and Developmental Neurobiology, Cajal Institute, Consejo Superior de Investigaciones Científicas, 28002 Madrid, Spain; 2 Tetraneuron, 28002 Madrid, Spain

**Keywords:** E2F4, Phosphorylation, Homeostasis, Camptothecin, Cell stress, Biochemistry, Cell biology, Molecular biology, Neuroscience

## Abstract

E2F4 is a transcription factor involved in cellular homeostasis and a substrate of the stress-activated kinase p38^MAPK^, which phosphorylates a conserved Thr248/Thr250 motif. A non-phosphorylatable mutant, E2F4DN (Thr248Ala/Thr250Ala), has demonstrated preclinical efficacy in a murine model of Alzheimer’s disease (AD), but its mechanism of action remains unknown. We hypothesized that cell stress-induced phosphorylation disrupts E2F4’s homeostatic function, whereas exogenous E2F4DN restores it. To begin testing this hypothesis, we treated differentiated N2a neuroblastoma cells (N2a neuron-like cells) with camptothecin (CPT) to induce genotoxic stress. CPT activated p38^MAPK^ within 8 h, leading to phosphorylation of E2F4 at Thr248/Thr250. We then overexpressed E2F4DN or a phosphomimetic variant, E2F4CA (Thr248Glu/Thr250Glu), and assessed apoptosis via procaspase-3 cleavage. The pro-apoptotic factor E2F1 strongly induced caspase-3 activation in this model system. This effect was partially mimicked by E2F4CA, while E2F4DN markedly suppressed it. Notably, E2F4DN, but not E2F4CA, upregulated the antiapoptotic factor Cited2, and knockdown experiments suggest it may contribute to E2F4DN’s protective effect. Overall, these findings indicate that E2F4DN counteracts p38^MAPK^-driven neuronal apoptosis and helps preserve neuronal homeostasis, at least in part, through Cited2 upregulation. This provides mechanistic insight into the neuroprotective role of E2F4DN as a potential therapy for AD.

**Supplementary Information:**

The online version contains supplementary material available at 10.1038/s41598-026-54592-z.

## Introduction

E2F4 is a member of the E2F family of transcription factors, which also includes E2F1, a canonical regulator of the G1/S phase transition in proliferative cells with capacity to induce cell death in differentiated neurons^1,2^. E2F4 was initially described as a cell cycle regulator crucial for the establishment of quiescence in proliferative cells^3^. Nevertheless, this transcription factor can regulate more than 7,000 genes^4^ that play important roles in cell differentiation, maintenance of DNA integrity, RNA processing, apoptosis, ubiquitination, and cell stress response. Therefore, E2F4 is currently acknowledged as a major player in cell and tissue homeostasis and tissue regeneration^5–8^.

There is growing evidence that E2F4 has a multifactorial impact in neuronal welfare and brain homeostasis^9,10^, and that this transcription factor might be involved in the physiopathology of neurodegenerative diseases, such as Alzheimer disease (AD), and brain aging^7,8^. E2F4 regulates most of AD-specific gene networks in humans^11^, and becomes upregulated in human AD neurons^12^ as well as in neurons derived from human induced pluripotent stem cells obtained from familial AD patients^13^. Furthermore, the expression of E2F4 is enriched in blood from mild cognitive impairment (MCI) and AD^14^. Finally, E2F4 participates in the protective action of memantine and donepezil against cognitive impairment induced by nine common endocrine-disrupting chemicals^15^.

The regulation of E2F4 is complex, as it can be post-translationally modified through acetylation and methylation^8^. In addition, E2F4 is susceptible to become phosphorylated by several protein kinases^16–18^, and the phosphorylation of E2F4 by the stress kinase p38^MAPK^ within an evolutionary-conserved threonine motif (Thr248/Thr250 in the human sequence and Thr249/Thr251 in mouse) can induce cell cycle re-entry in neurons^16^. This latter effect can be induced by a phosphomimetic form of E2F4 carrying Glu residues in substitution of the conserved threonines (referred to as E2F4CA), and inhibited by a dominant negative form of E2F4 (referred to as E2F4DN) carrying alanines instead of the conserved Thr residues^16^.

There is indirect evidence that the Thr conserved motif of E2F4 can be phosphorylated in AD-affected human neurons, based on an E2F4-specific proximity-ligation assay (PLA)^10^. Moreover, a phospho-Thr249-specific antibody generated in our laboratory was able to detect the phosphorylated form of E2F4 in the brain of homozygous 5xFAD mice^9^, a known murine model of Alzheimer’s disease (AD)^19^ with aggravated phenotype due to the increase of gene dosage^20^. E2F4 phosphorylation at this conserved motif is not specific for the nervous system. Thus, the phosphorylation of Thr248 in human E2F4 and of Thr249 in the mouse sequence has been detected, in retinal pigment epithelium-derived cells and in embryonic stem cells, respectively^18^.

Preclinical studies in homozygous 5xFAD mice have demonstrated that E2F4DN is a safe and effective multifactorial therapeutic agent, providing a strong rationale for its progression to clinical trial application. E2F4DN administration in these mice led to a reduction in amyloid deposition, gliosis, inflammation, neuronal death, oxidative stress, and neuronal tetraploidization^8,9,10^, while also preventing dendritic and synaptic alterations and preserving synaptic long-term potentiation^21^. It also extended the life span in both wild-type (WT) and 5xFAD mice, prevented body weight loss and promoted fur regeneration in senile mice^8–10^.

Our current hypothesis for the mechanism of action of E2F4DN is that the phosphorylation of the Thr248/Thr250 motif by the stress kinase p38^MAPK^ prevents the homeostatic function of endogenously-expressed E2F4, while the presence of exogenous E2F4DN can restore this function. In this study, we have begun testing this hypothesis by focusing on mouse Neuro-2a (N2a) neuroblastoma cells^22^ cultured under low serum conditions to induce neuronal differentiation. These N2a neuron-like cells (N2a NCs) were treated with camptothecin (CPT) to induce DNA damage, as a known mechanism of cell stress followed by cell death in neurons^23^. This effect is potentiated by E2F1 expression in a cell cycle-independent manner^2,24,25^, while E2F4 can regulate E2F1 function in this cellular stress paradigm^26^. We found that, under these conditions, p38^MAPK^ activity becomes activated in N2a NCs, and this activation correlates with the phosphorylation of endogenous mouse E2F4 at Thr249, an effect that is prevented by the p38^MAPK^-specific inhibitor SB203580. The significance of E2F4 phosphorylation in apoptosis was tested by overexpressing either the phosphomimetic form of E2F4 (E2F4CA) or E2F4DN, followed by quantification of procaspase-3 cleavage and pyknosis as readouts of apoptosis. E2F1, which was used as a positive control, induced a statistically-significant increase of cleaved caspase-3 48 h after CPT treatment. This was partially replicated by E2F4CA, while E2F4DN expression inhibited this effect. Finally, we provide evidence that Cbp/P300-interacting transactivator with Glu/Asp-rich carboxy-terminal domain 2 (Cited2) may be required for the antiapoptotic effect of E2F4DN in this cellular paradigm. We conclude that the phosphorylation of E2F4 prevents its survival effect in N2a NCs subjected to genotoxic stimuli, while E2F4DN rescue these cells from apoptosis. This finding provides support to the hypothesis that neuronal expression of E2F4DN maintains the homeostatic function of E2F4 under cell stress conditions.

## Results

### Low serum concentration favors neuronal differentiation of N2a neuroblastoma cells

To study the role of E2F4 phosphorylation in neuronal DNA damage response we focused on mouse N2a neuroblastoma cells due to the capacity of this cell line to differentiate as neurons under low serum conditions^27–30^. N2a cells were therefore cultured in the presence of 1% fetal bovine serum (FBS) to induce neuronal differentiation^30^.

Under these conditions, most N2a cells showed processes resembling dendrites (Supplementary Fig. 1A), which were immunopositive for Microtubule-associated Protein 2 (MAP2) (Supplementary Fig. 1B), a protein that is enriched in dendrites and cell bodies^31^ as well as in proximal axons^32^. Moreover, these N2a NCs expressed both *Syn1*-specific mRNA (Supplementary Fig. 1C) and Synapsin1 protein (Supplementary Fig. 1B). Nevertheless, in contrast to a former study^33^, which reported increased Syn1 expression in N2a neuroblastoma cells following retinoic acid–induced differentiation, our differentiation protocol is mechanistically distinct. We observed that this protein and its coding mRNA was expressed at similar levels in both undifferentiated N2a neuroblastoma cells and N2a NCs (Supplementary Fig. 1B,C). The capacity of N2a NCs to express Synapsin 1 makes these cells an ideal system to express exogenous proteins under the control of the human synapsin1 (*hSyn1*) promoter^34^.

### Expression of E2F4 and its mutant forms E2F4DN and E2F4CA in N2a NCs

**Fig. 1:**
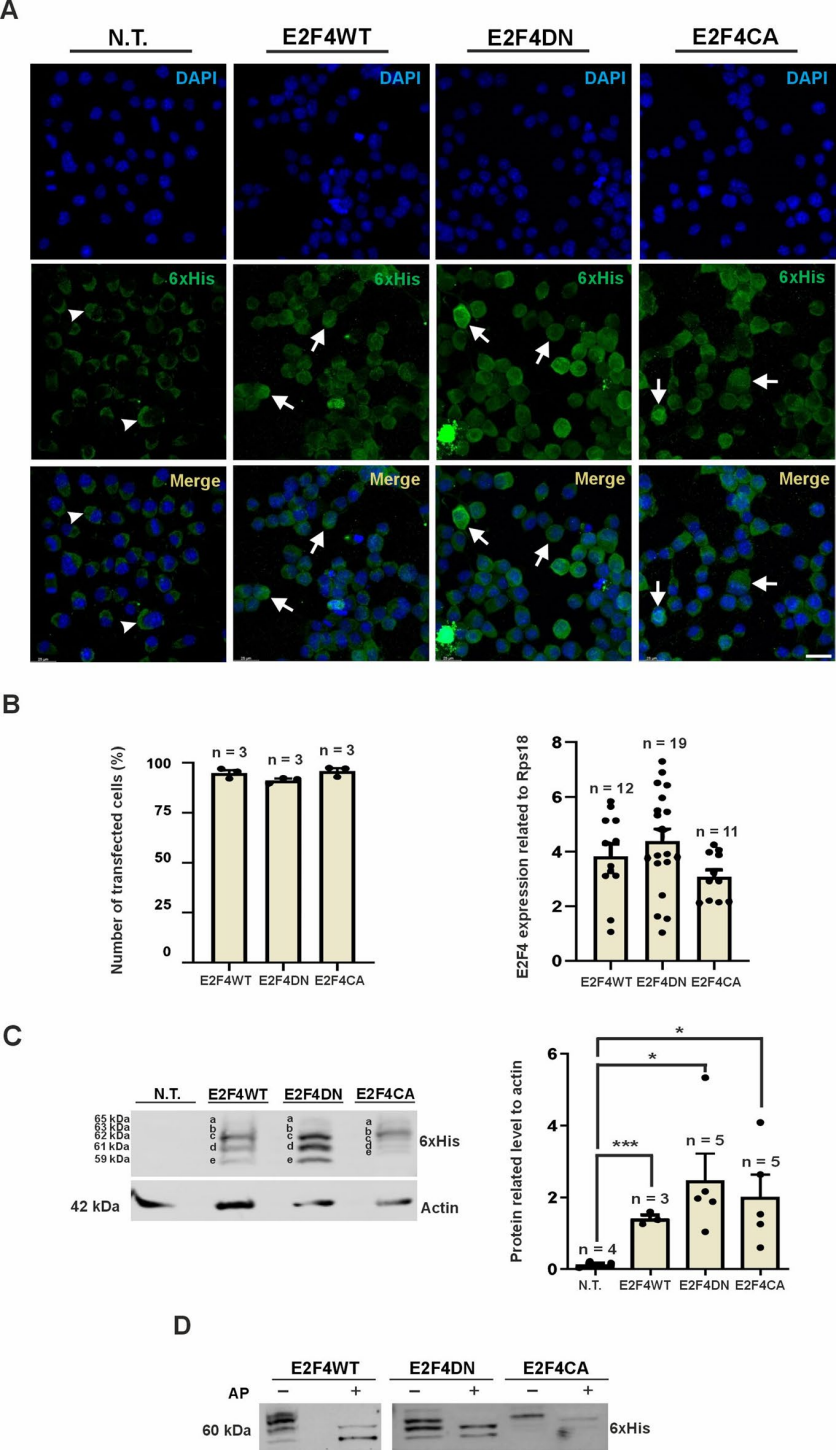
Overexpression and phosphorylation of different E2F4 forms in N2a NCs. (**A**) Representative inmunostaining of N2a NCs, either non-transfected (N.T.) or transduced with Ad5 vectors expressing E2F4WT-6xHis (E2F4WT), E2F4DN-6xHis (E2F4DN) or E2F4CA-6xHis (E2F4CA), using an anti-6xHis antibody (green). Notice that, while unspecific fluorescence can be observed in the non-transfected cells (arrowhead), characterized by a crescent-like structure that is not observed in transduced cells, 6xHis-specific labeling with different intensity levels was observed in the transfected cells (arrows). Cell nuclei were labeled with DAPI (blue). Bar: 25 μm. (**B**) Left: Quantification of E2F4WT-6xHis, E2F4DN-6xHis and E2F4CA-6xHis transduction efficiency in the N2 neuronal cultures shown in (**A**) (i.e. number of 6xHis^+^ cells related to DAPI^+^ nuclei). Right: Quantification by qRT-PCR of the DNA constructs expressing E2F4WT, E2F4DN, and E2F4CA. (**C**) Left panel: representative western blot of the multiband pattern (bands a-e) observed in cell extracts from E2F4WT-6xHis, E2F4DN-6xHis or E2F4CA-6xHis-transduced N2a NCs, as revealed with an anti-6xHis antibody. Actin was used as a loading marker. Right panel: quantification of the intensity of all bands shown in the left panel relativized to actin. n: number of independent experiments. *p < 0.05; **p < 0.01 (Kruskal–Wallis test followed by a corrected Dunn post hoc test). (**D**) Representative western blots of the effect of alkaline phosphatase (AP) on the multiband pattern observed in cell extracts from E2F4WT-6xHis-, E2F4DN-6xHis- or E2F4CA-6xHis-transduced N2a NCs. Note that the bands shown were grouped from cropped blots obtained from different gels. Specifically: C (upper), box 1 in Supplementary Material Fig. 1; C (lower), box 2 in Supplementary Material Fig. 1; D (left), box 3 in Supplementary Material Fig. 1; D (right) box 4 in Supplementary Material Fig. 1. White spaces were inserted to delineate the different cropped blots.

We confirmed by quantitative reverse transcriptase PCR (qRT-PCR) that N2a NCs were able to express E2F4WT and its mutant forms E2F4DN and E2F4CA, driven by the *hSyn1* promoter (Fig. 1B, right panel). By using an anti-6xHis specific antibody, we verified by immunocytochemistry that E2F4WT-6xHis, E2F4DN-6xHis and E2F4CA-6xHis are widely expressed in N2a NCs 48 h after transduction with the Ad5.E2F4WT-6xHis, Ad5.E2F4DN-6xHis, and Ad5.E2F4CA-6xHis vectors, respectively (Fig. 1A). Non-transduced control cells exhibited unspecific labeling that allowed us to set up a threshold above which cells were classified as positive (Supplementary Fig. 2). Quantification of 6xHis-positive cells based on this threshold indicated that over 95% of N2a NCs expressed the constructs (Fig. 1B, left panel). Moreover, western blot (WB) analysis with the anti-6xHis antibody further confirmed the expression of these proteins (Fig. 1C). Interestingly, the different forms of E2F4 displayed a multiband pattern (Fig. 1C). This suggests that, in N2a NCs, E2F4 can be phosphorylated in multiple residues, as previously described in other cell types^17,35^. As in these later studies, we found five different bands for this transcription factor (bands *a* to *e*), with different mobility. As expected, the phosphomimetic nature of E2F4CA led to the presence of lower mobility bands, which were also named from *a* to *e*, being bands *b* and c those with the highest intensity (Fig. 1C).

The incubation of the cell extracts with alkaline phosphatase (AP) demonstrated that most of the bands that were observed in E2F4WT-, E2F4DN-, and E2F4CA-expressing N2a NCs derive from different phosphorylation states of the molecule. In this regard, only two high mobility bands (*d* and *e*) remained in the presence of this enzymatic activity (Fig. 1D), suggesting that one of these two bands contains a post-translational modification different from phosphorylation^8^. This analysis was intended to be qualitative, as band intensities were not quantified.

### Dynamic localization of E2F4WT, E2F4DN and E2F4CA in subcellular domains

**Fig. 2:**
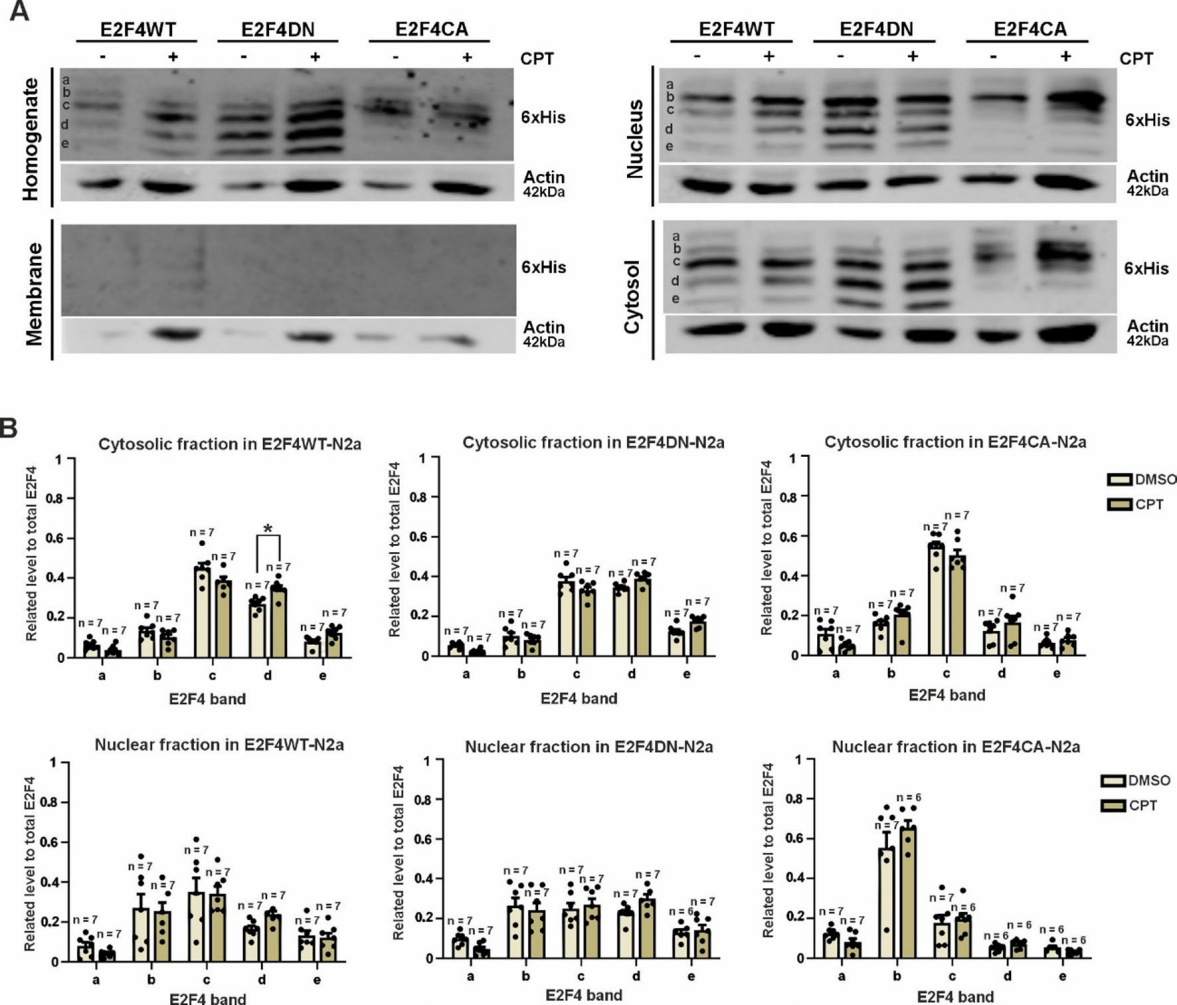
Subcellular localization of the different phosphorylation forms of E2F4 in N2a NCs. (**A**) Western blots of the multiband pattern detected in the indicated homogenates and subcellular fractions from E2F4WT-6xHis- (E2F4WT), E2F4DN-6xHis- (E2F4DN) or E2F4CA-6xHis-transduced N2a NCs (E2F4CA), either under control conditions or 8 h after CPT treatment, as revealed with an anti-6xHis antibody. Actin was used as a loading marker. MWs as those shown in Fig. 1. In the whole homogenate, the bands with higher intensity are c (E2F4WT), c and d (E2F4DN), and c (E2F4CA), corresponding to those enriched in the cytosolic fraction. In contrast, band b, which is enriched across all nuclear fraction lanes, is underrepresented in the homogenate, consistent with the staining pattern shown in Fig. 1A. Note that the images are not fully representative of the quantification shown in (**B**), due to the high degree of variability among band intensities. (**B**) Quantification of the relative frequency of the bands a-e that are present in the cytosolic and nuclear fractions for each experimental condition (see panel A). Note that the bands shown were grouped from cropped blots obtained from different gels. Specifically: A (left, upper), boxes 5 and 8 in Supplementary Material Fig. 1; A (left, lower), boxes 11 and 12 in Supplementary Material Fig. 1; A (right, upper), boxes 7 and 10 in Supplementary Material Fig. 1; A (right, lower), boxes 6 and 9 in Supplementary Material Fig. 1. n: number of independent experiments. *p < 0.05 [Two-way ANOVA, followed by Tukey’s honestly significant difference (HSD) post-hoc test].

To analyze the distribution of the different E2F4 bands across cellular compartments, we prepared membrane, cytosolic, and nuclear fractions from N2a NCs expressing exogenous E2F4WT, E2F4DN, or E2F4CA. This analysis indicated that, as expected from a soluble protein, all forms of E2F4 were present in the cytosolic and nuclear compartments while E2F4 was essentially absent from the membrane fraction (Fig. 2A). The pattern of band intensities differed between the nuclear fraction and the cytosolic fraction. Bands *c* and *d* were the main phosphorylated states observed in the cytosol for both E2F4WT and E2F4DN, while E2F4CA, in accordance with its phosphomimetic nature, displayed higher levels of phosphorylation in this subcellular compartment as its main band was *c* (Fig. 2B). In contrast, a shift towards bands with increased phosphorylated levels was detected in the nucleus (particularly band *b*), with different degrees depending on the specific form of E2F4 that was analyzed. Nuclear E2F4CA was mainly in the form of band *b*, while the predominant state of E2F4WT was band *c*. Finally, bands *b*, *c*, and *d* were equally displayed for nuclear E2F4DN, indicative of its reduced capacity to become phosphorylated due to the Thr248Ala/Thr250Ala mutation. The differences of phosphorylation state of E2F4DN and E2F4CA (i.e. higher phosphorylation in E2F4CA vs lower phosphorylation in E2F4DN) suggest that the function of E2F4 as a transcription factor, but also as a homeostatic protein^8^, may differ depending on the phosphorylation state of this latter Thr motif. The consistent and reproducible distribution pattern of the E2F4-specific bands, with well-defined intensities in the nuclear and cytosolic fractions and no detectable signal in the membrane fraction, supports the reliability of the subcellular fractionation and is consistent with the pattern of established compartment-specific markers in each fraction (Supplementary Fig. 3). The purity of the cytosolic fraction was confirmed by the presence of a GAPDH band at ~ 36 kDa. The nuclear fraction contained a ~ 29-kDa PCNA band. In contrast, the membrane fraction specifically contained the α1 subunit of the Na^+^/K^+^ ATPase (Na^+^/K^+^ ATPase α1) at ~ 112 kDa. Together, these results confirm the enrichment and minimal cross-contamination of the subcellular fractions. This analysis was intended to be qualitative, as band intensities were not quantified.

To study whether DNA damage may induce specific E2F4 phosphorylation at the Thr248/Thr250 motif, we focused on N2a NCs treated with 10 µM CPT as a paradigm of DNA damage^36^. The presence in the culture medium of this topoisomerase I inhibitor for 8 h did not substantially modified the phosphorylation profile, with the exception of band *d* in the cytosolic fraction from E2F4WT-expressing N2a NCs, which significantly increased its intensity in the presence of CPT (Fig. 2B). The intensity of this band was not significantly altered in the cytosolic fraction of both E2F4DN- and E2F4CA-expressing N2a NCs, suggesting that it results from the phosphorylation of the Thr248/Thr250 motif of E2F4 in response to CPT treatment.

### CPT treatment induces p38^MAPK^ activity in N2a NCs

**Fig. 3:**
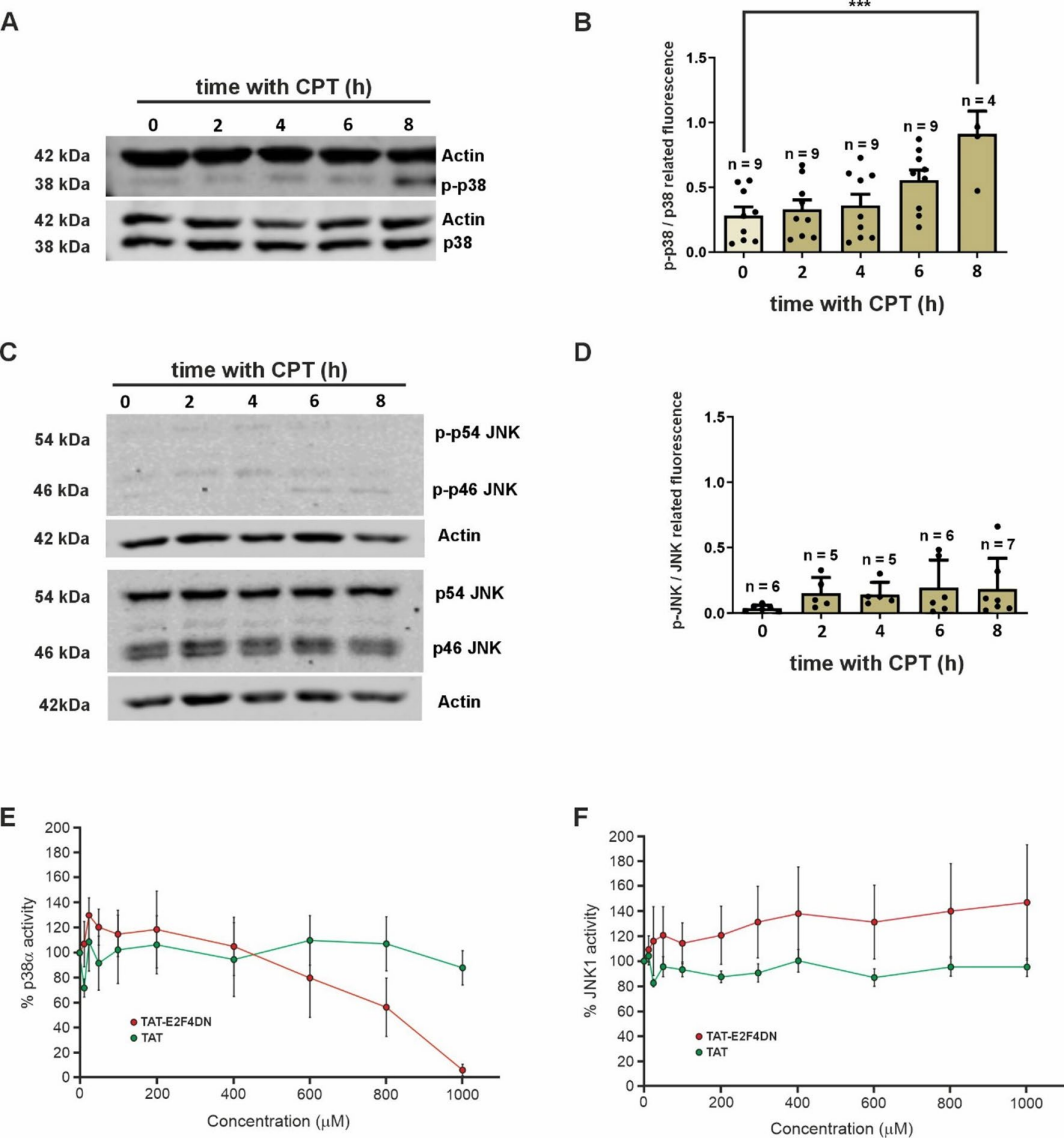
p38^MAPK^ activation in response to CPT in N2a NCs. (**A**) A representative western blot from cell extracts of N2a NCs isolated at the indicated time points after 10 µM CPT treatment, using an antibody recognizing active p38^MAPK^ (p-p38) and total p38^MAPK^ (p38). Actin was used as a loading marker. (**B**) Quantification of p38^MAPK^ activation as the ratio between active p38^MAPK^ (relativized to Actin) and total p38^MAPK^ (relativized to Actin) at the indicated time points after CPT treatment. n: number of independent experiments. ***p < 0.001 (One-way ANOVA, followed by Tukey’s HSD post-hoc test). (**C**) Representative western blot from cell extracts of N2a NCs isolated at the indicated time points after 10 µM CPT treatment, using an antibody recognizing active JNK (p-p54 JNK and p-p46 JNK) and total JNK (p54 JNK and p46 JNK). Actin was used as a loading marker. (**D**) Quantification of JNK activation as the ratio between the sum of active JNK (p-p54 JNK plus p-p46 JNK relativized to Actin) and total JNK (p54 JNK plus p46 JNK relativized to Actin) at the indicated time points after CPT treatment. (**E**) A p38^MAPK^ kinase assay performed in the presence of either a control peptide (TAT) or a TAT-E2F4DN peptide at the indicated concentrations (TAT: n = 3 independent experiments, each with 3 technical replicates per condition; TAT-E2F4DN: n = 4 independent experiments, each with 3 technical replicates per condition). (**F**) A JNK assay performed in the presence of either a control peptide (TAT) or a TAT-E2F4DN peptide at the indicated concentrations (n = 2 independent experiments, each with 3 technical replicates per condition). Note that the bands shown were grouped from cropped blots obtained from different gels. Specifically: A (upper), box 14 in Supplementary Material Fig. 1; A (lower), box 13 in Supplementary Material Fig. 1; C (upper), box 15 in Supplementary Material Fig. 1; C (middle, upper), box 17 in Supplementary Material Fig. 1; C (middle lower), box 16 in Supplementary Material Fig. 1; C (lower), box 18 in Supplementary Material Fig. 1.

In chick neurons, the stress kinase p38^MAPK^ can phosphorylate E2F4 in the Thr265/Thr267 conserved domain^16^, which is orthologous of Thr248/Thr250 in human E2F4 and Thr249/Thr251 in mouse E2F4 (notice that we will refer to Thr248/Thr250 in those experiments where the different variants of human E2F4 are expressed using Ad5 vectors, whereas the term Thr249/Thr251 will be used for those experiments where the phosphorylation state of endogenous mouse E2F4 is evaluated). In a mouse model of AD, E2F4 has been shown to become phosphorylated in Thr249 (9), likely being this phosphorylation part of the etiopathology of the disease, which includes p38^MAPK^ activation at its early stages^37–40^. These observations suggest that stressful conditions triggering p38^MAPK^ activation may lead to p38^MAPK^-dependent phosphorylation of E2F4. To directly test the hypothesis that E2F4 can be phosphorylated by p38^MAPK^ in the Thr249/Thr251 motif, N2a NCs were treated with 10 µM CPT to induce DNA damage, as a mechanism of cell stress followed by cell death in neurons^23^. We found that, under these conditions, p38^MAPK^ activity becomes gradually activated, an effect that is already evident at 6 h while showing statistically significant differences 8 h after CPT treatment (Fig. 3B). This effect, which was based on the dual phosphorylation of p38^MAPK^ on Thr180 and Tyr182^41^, was specific for p38^MAPK^ since the levels of active c-Jun N-terminal kinases (JNK) were not significantly upregulated at the studied time points (Fig. 3D).

### p38^MAPK^ specifically phosphorylates Thr249 in endogenous E2F4

To verify that the activation of p38^MAPK^ in N2a NCs treated with 10 µM CPT results in the phosphorylation of endogenous E2F4 in its Thr249/Thr251 motif, we used an antibody that recognizes the phosphoThr249 epitope of mouse E2F4, previously generated and characterized in our laboratory^9^. The specificity of this antibody was further confirmed by a direct ELISA assay, which showed preferential recognition of a Thr249-phosphorylated E2F4 peptide over the corresponding non-phosphorylated control peptide (Supplementary Fig. 4).

**Fig. 4:**
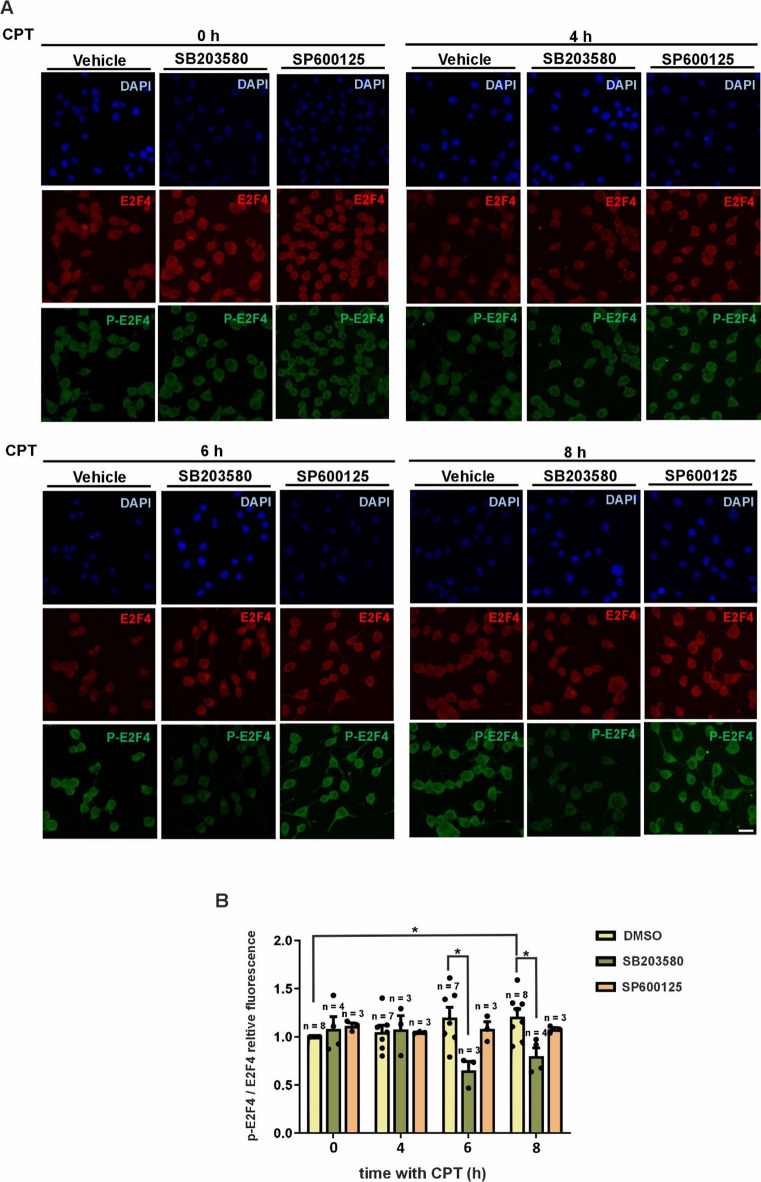
E2F4 phosphorylation at Thr249 in N2a NCs treated with CPT. (**A**) Representative immunostaining of N2a NCs treated with 10 µM CPT for the indicated time points in the presence of vehicle, the p38^MAP^ inhibitor SB203580 or the JNK inhibitor SP600125, using antibodies specific for total E2F4 (E2F4) or E2F4 phophorylated at Thr249 (P-E2F4). Cell nuclei were labeled with DAPI (blue). Bar: 25 μm. (**B**) Quantification of endogenous E2F4 phosphorylation at Thr249 of in response to 10 µM CPT treatment by measuring the ratio between p-E2F4 and E2F4 fluorescence intensities for each experimental condition. n: number of independent experiments. *p < 0.05 (Two-way ANOVA, followed by Tukey’s HSD post-hoc test).

N2a NCs were cultured in the presence of 10 µM CPT for 0 (untreated control cells), 4, 6 and 8 h. Under these conditions, E2F4 expression levels were increased (Supplementary Fig. 5). These cultures were then fixed and immunostained with the phospho-specific anti-E2F4 antibody together with an antibody recognizing E2F4 independently of its phosphorylation state, and then the phosphoE2F4/E2F4 ratio was calculated for each experimental condition. We found that the phospho-E2F4/E2F4 ratio was significantly higher in N2a NCs treated with CPT for 8 h (Fig. 4A, B), in accordance with the significant activation of p38^MAPK^ at this time point (see above). This result is consistent with the significant increase of the band *d*, observed specifically in the cytosol of E2F4WT-, but not E2F4DN- or E2F4CA-expressing N2a NCs treated for 8 h with CTP (Fig. 2A, B).

To test whether the Thr249-specific phosphorylation of E2F4 in response to CPT was associated to p38^MAPK^ activity, N2a NCs were cultured in the same conditions as above either in the presence of 10 µM SB203580, a p38^MAPK^-specific inhibitor^42,43^, or in the presence of 25 µM SP600125, a JNK-specific inhibitor^44^, used as a pathway control. Both inhibitors have been previously validated in N2a cells^45–47^. This analysis indicated that the presence of SB203580, but not SP600125, was able to significantly reduce the phosphorylation of E2F4 at both 6 and 8 h (Fig. 4A, B), the time points at which p38^MAPK^ activation in response to the CTP treatment was already evident (Fig. 3A, B).

A cell-free kinase assay was performed to confirm the capacity of p38^MAPK^ to functionally interact with E2F4. By incubating a p38^MAPK^-specific substrate together with ATP and activated p38α, we were able to show dose-dependent inhibition of the p38^MAPK^ activity using a mouse E2F4-derived peptide containing the Thr249Ala/Thr251Ala mutation (Fig. 3E). In contrast, this same peptide was not able to prevent the JNK activity when tested in a cell-free JNK1 kinase assay (Fig. 3F). These experiments provide direct biochemical evidence supporting a selective p38^MAPK^–E2F4 interaction through the Thr249/Thr251 conserved motif, known to become phosphorylated in chicken E2F4^16^.

### E2F4DN expression prevents CPT-induced cell death in N2a NCs

CPT treatment is known to induce apoptosis^23^, which is potentiated by E2F1 expression in a cell cycle-independent manner^2,24,25^. Therefore, we performed a temporal analysis to evaluate the time point at which N2a NCs overexpressing E2F1 start to die in response to CPT, as evidence by caspase-3 activation. This analysis indicated that a statistically significant increase of apoptosis can be detected in these cells 48 h after CPT treatment (Supplementary Fig. 6).

**Fig. 5:**
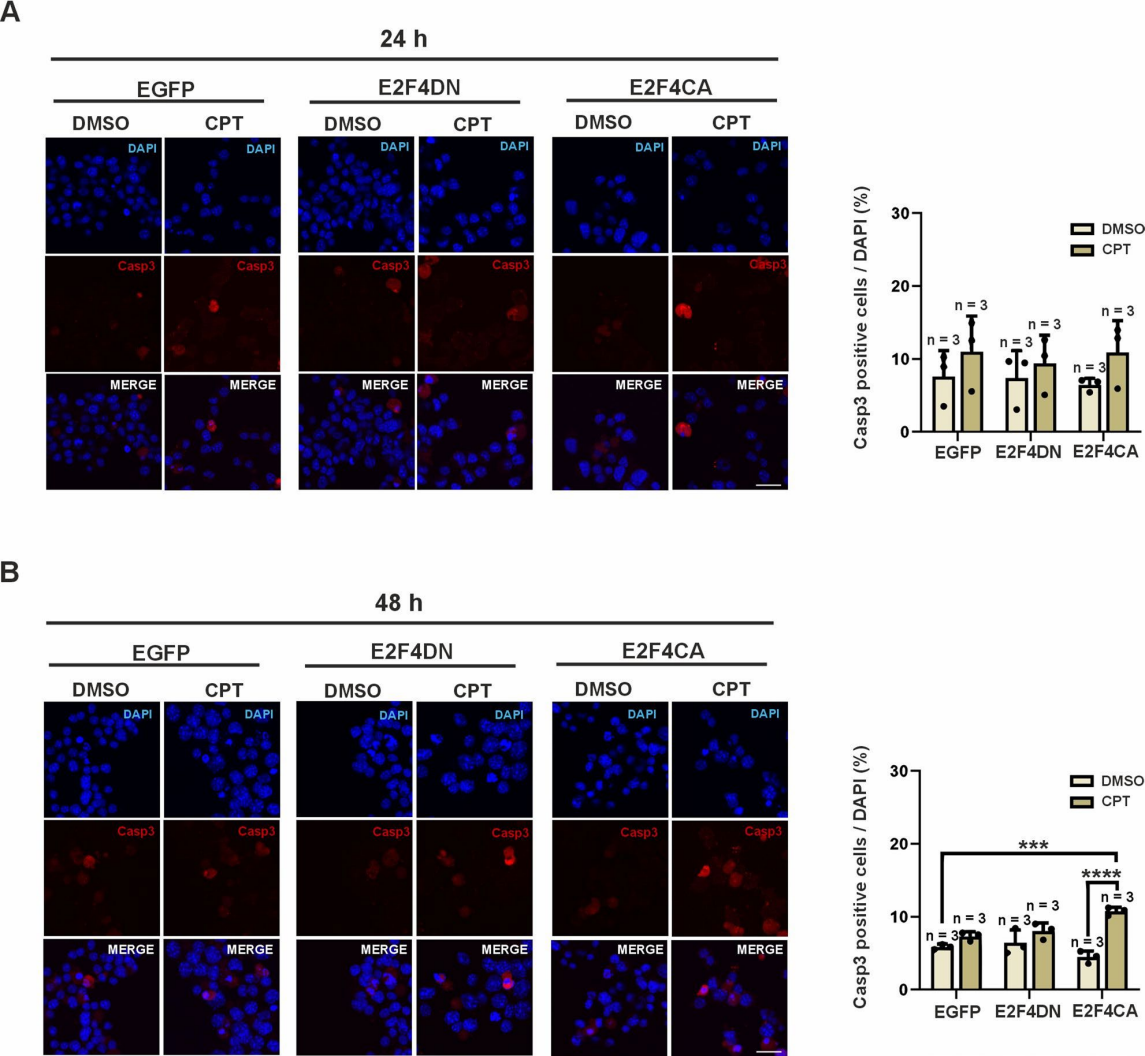
Regulation of apoptosis by E2F4 phosphorylation in CPT-treated N2a NCs. Left panels: Representative active caspase-3 immunostaining (red) of N2a NCs transduced with the indicated forms of E2F4 and treated with 10 µM CPT for 24 h (**A**) or 48 h (**B**). Cell nuclei were labeled with DAPI (blue). Right panels: Percentage of active caspase-3-positive N2a NCs related to total DAPI-positive nuclei for each indicated experimental condition. n: number of independent experiments. ***p < 0.005; ****p < 0.0001 (Two-way ANOVA, followed by Tukey’s HSD post-hoc test). Bar: 25 μm.

CPT treatment can lead to the degradation of Rb protein and phosphorylation of its family members, p107 and p130^48,49^. Therefore, the release of E2F4 may participate in CPT-induced apoptosis, as all these pocket protein members are known E2F4 interactors^50^. To test whether E2F4 and its phosphorylation at the Thr248/Thr250 motif participates in CPT-induced apoptosis, we cultured N2a NCs transduced with E2F4CA, followed by CPT treatment for 24 h or 48 h. This analysis indicated that, like E2F1, E2F4CA was able to induce a statistically significant increase of apoptosis, quantified as the proportion of cells containing active caspase 3 (Fig. 5A,B) and pyknotic nuclei (Supplementary Fig. 7), in N2a NCs when co-treated with 10 μM CPT for 48 h, but not for 24 h (Fig. 5A,B). In contrast, no significant induction of apoptosis was observed in these cells in the presence of E2F4DN (Fig. 5A,B). Therefore, the phosphorylation of E2F4 in its Thr248/Thr250 motif is likely required for the trigger of apoptosis by CPT.

### The antiapoptotic effect of E2F4DN in N2a NCs is independent of DNA repair

**Fig. 6:**
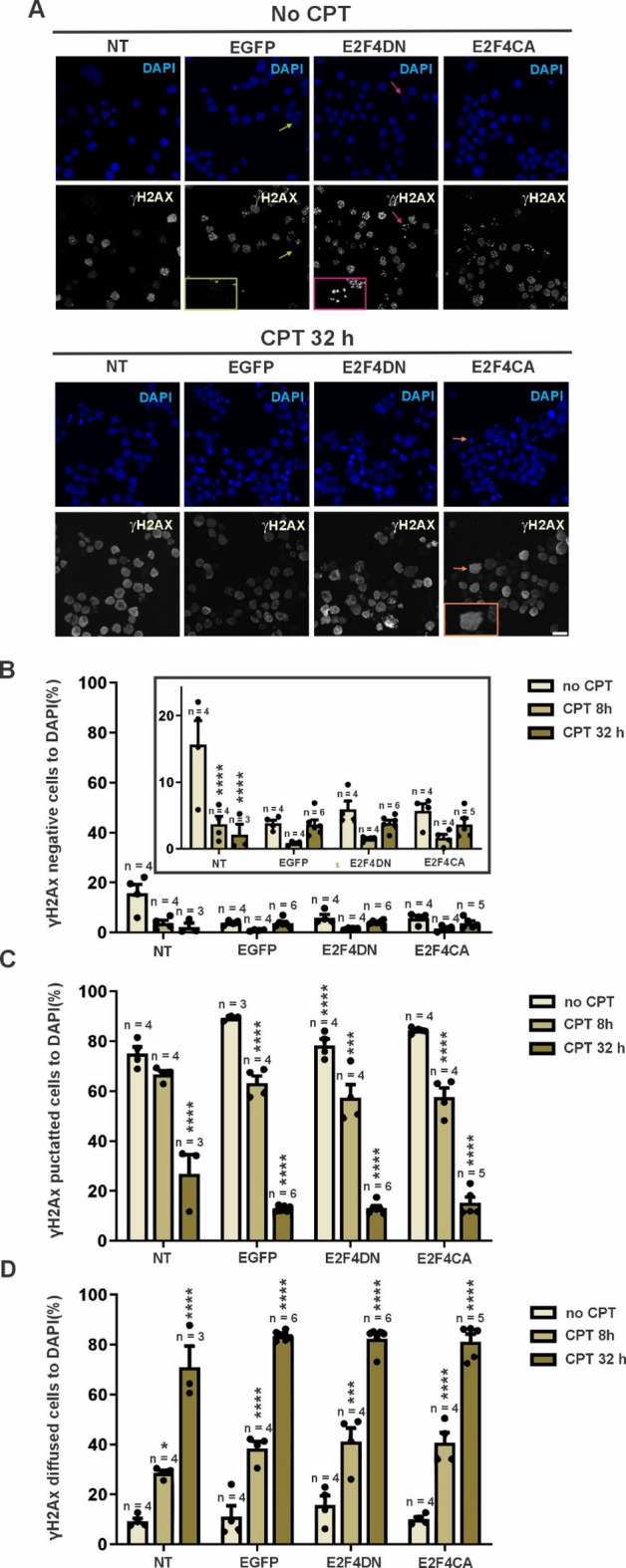
DNA damage regulation by E2F4 in N2a NCs. (**A**) Representative inmunostaining performed with an antibody specific for γH2AX to identify double strand breaks in the genomic DNA of N2a NCs transduced with either EGFP (EGFP), E2F4DN-6xHis (E2F4DN) or E2F4CA-6xHis (E2F4CA) and treated with 10 µM CPT for 32 h or left untreated (NT). Orange arrow: a γH2AX-positive cell with diffuse staining shown at high magnification in the orange box. Green arrow: a γH2AX-negative cell shown at high magnification in the green box. Pink arrow: a γH2AX-positive cell with punctate staining shown at high magnification in the pink box. White labeling was obtained using secondary antibodies conjugated to Alexa Fluor 647. Bar: 25 μm. (**B**) Quantification of the γH2AX-negative N2a NCs, related to DAPI-positive nuclei, for each experimental group. (**C**) Quantification of the γH2AX-positive N2a NCs showing punctate staining, related to DAPI-positive nuclei, for each experimental group. (**D**) Quantification of the γH2AX-positive N2a NCs showing diffuse staining, related to DAPI-positive nuclei, for each experimental group. n: number of independent experiments. ***p < 0.005, ****p < 0.001 (Two-way ANOVA, followed by Tukey’s HSD post-hoc test).

DNA damage is known to trigger apoptosis, mainly through a p53-dependent mechanism^51^ although it may also rely on the transfer RNAse SLFN11 in p53 mutant cells^52^. Therefore, the antiapoptotic effect of E2F4DN in CPT-treated N2a NCs might depend primarily on the potential effect of E2F4 to favor DNA repair since it regulates the expression of DNA repair genes^8^. Alternatively, E2F4 might prevent apoptosis in these cells by directly acting on the proapoptotic machinery downstream of DNA damage. To test whether E2F4DN prevents DNA damage in response to CPT treatment prior to the detection of apoptosis, N2a NCs, previously transduced with EGFP, E2F4DN or E2F4CA, or left untreated, where maintained for either 0, 8, or 32 h in the presence of vehicle or of 10 μM CPT. Then, cultures were fixed and immunostained with an anti-γH2AX antibody to identify double strand DNA breaks^53^. This analysis demonstrated that DNA damage is increased with time for all the experimental conditions that were analyzed, as revealed by the γH2AX-specific labeling. Indeed, while the proportion of cells with punctate γH2AX labeling (i.e. cells with low DNA damage levels) gradually decreased with time (Fig. 6A, C), those showing a diffuse γH2AX pattern (i.e. cells with strong DNA damage) progressively increased its proportion with time (Fig. 6A, D). Interestingly, no statistically significant differences were observed at any time point in the proportion of CPT-treated N2a NCs either lacking γH2AX-specific immunostaining or showing punctate or diffuse γH2AX labeling when E2F4DN or E2F4CA were expressed as compared to control cells (Fig. 6B-D). Therefore, our results indicate that the antiapoptotic effect of E2F4DN in response to CPT-induced DNA damage likely relies on its interaction with the downstream pathways involved in cell death.

### Cited2 acts downstream of E2F4DN to prevent CPT-dependent N2a neuronal death

**Fig. 7:**
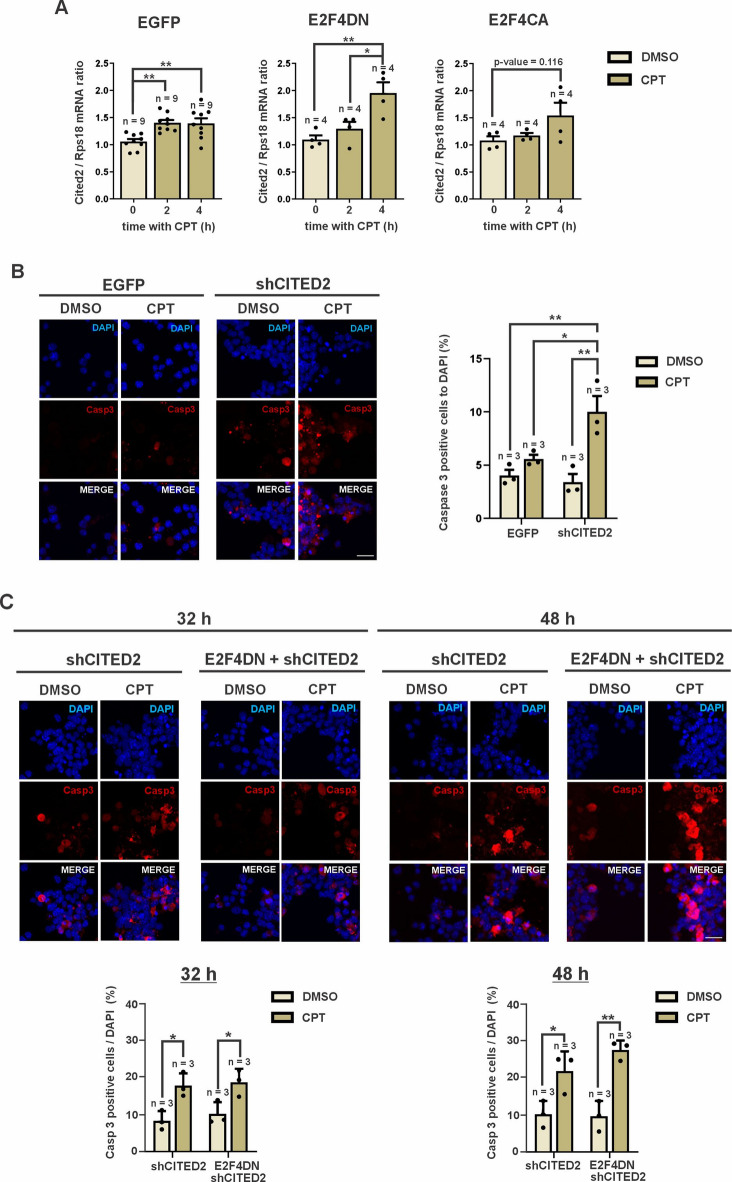
Participation of Cited2 in the CPT-induced apoptotic cell death regulated by E2F4 in N2a NCs. (**A**) Normalized quantification of *Cited2* mRNA levels by qRT-PCR, normalized to the *Rps18* housekeeping gene, in cDNA obtained from N2a NCs transduced with either EGFP (EGFP), E2F4DN-6xHis (E2F4DN) or E2F4CA-6xHis (E2F4CA) treated for the indicated time periods with 10 µM CPT. *p < 0.05; **p < 0.01 (One-way ANOVA). (**B**) Percentage of active caspase-3-positive N2a NCs transduced with either EGFP or a *Cited2*-specific shRNA (shCITED2) and cultured for 32 h with 10 µM CPT, related to total DAPI-positive nuclei. (**C**) Percentage of active caspase-3-positive N2a NCs transduced with either 60 IU/cell of an Ad5 vector expressing a *Cited2*-specific shRNA plus 60 IU/cell of an Ad5.EGFP vector (shCITED2), or 60 IU/cell of an Ad5.E2F4DN-6xHis vector plus 60 IU/cell of an Ad5 vector expressing a *Cited2*-specific shRNA (E2F4DN/shCITED2), and cultured for 32 h or 48 h with 10 µM CPT, related to total DAPI-positive nuclei. Notice that the overall levels of apoptosis are higher than those observed in panel B, likely due to the double Ad5 vector transduction protocol. n: number of independent experiments. *p < 0.05; **p < 0.01 (Two-way ANOVA, followed by Tukey’s HSD post-hoc test). Bar: 25 μm.

Cited2 is a transcriptional regulator that can be regulated by the crosstalk of E2F1 and E2F4 in response to CPT treatment^26^. Depending on the cellular context, this protein can act either as an antiapoptotic factor^54,55^, since it can prevent p53 activation^56^, or as a proapoptotic agent^57,58^. Therefore, we hypothesized that Cited2 participates in the control of CPT-dependent cell death by E2F4DN due to the capacity of the latter to induce Cited2 expression. This hypothesis was tested using E2F4DN- and E2F4CA-expressing N2a NCs treated for 4 h with CPT to avoid any interference with active p38^MAPK^ since, at this time point, p38^MAPK^ has not yet been activated (see above). This analysis demonstrated that E2F4DN upregulates *Cited2* gene expression 4 h after CPT treatment when compared to both 0 h (untreated control) and 2 h. This contrasts with the inability of E2F4CA to significantly induce *Cited2* expression at this time point (Fig. 7A), thus indicating that Thr248/Thr250 phosphorylation prevents the capacity of E2F4 to express *Cited2*. Under control conditions (EGFP-expression), *Cited2* expressions was slightly upregulated 2 h after CPT treatment, but its levels of expression did not increase at the 4 h time point. To determine whether the differences observed upon CPT exposure reflected genuine treatment effects rather than pre-existing transcriptional differences, we examined baseline (0 h) Cited2 mRNA levels prior to normalization. Under control conditions, cells expressing E2F4DN displayed significantly lower Cited2 expression compared to cells expressing E2F4CA, while EGFP-transfected and non-transfected cells showed intermediate levels (Supplementary Fig. 8). Importantly, although baseline differences were present, the distinct response to CPT treatment observed in E2F4DN-expressing cells—opposite to that observed under basal conditions—indicates that the effect reflects a bona fide CPT-dependent regulatory response.

To test the participation of Cited2 in the prevention by E2F4DN of CPT-dependent cell death, we used an adenoviral vector carrying a *Cited2*-specific shRNA (Ad5-m-CITED2-shRNA). The capacity of this vector to co-express EGFP, allowed us to verify that ~ 75% of N2a NCs were transduced (Supplementary Fig. 9A). As expected, this shRNA was able to downregulate the expression of endogenous *cited2* mRNA in N2a NCs (Supplementary Fig. 9B). The transduction of N2a NCs with the Ad5-m-CITED2-shRNA vector indicated that Cited2 seems to have an antiapoptotic role in N2a NCs when treated with CPT (Fig. 7B). Therefore, the reduction of cell death that is observed in CPT-treated N2a treated with E2F4DN might derive, at least partially, from the capacity of the latter to upregulate *Cited2* expression. This view is consistent with the lack of antiapoptotic capacity of E2F4DN in CPT-treated N2a NCs in which Cited2 has been knockdown with the CITED2-shRNA construct at both 32 h and 48 h (Fig. 7C).

To address the possibility of off-target effects associated with the initial shRNA construct, we generated a second independent Ad5-m-CITED2-shRNA vector. This Ad5 vector also co-expressed EGFP, allowing the assessment of transduction efficiency, which reached 92.78 ± 0.49% in N2a NCs. Quantitative analysis confirmed a robust reduction of endogenous Cited2 mRNA levels (Supplementary Fig. 10A).

In contrast to the first shRNA depletion of *Cited2* using this second construct did not significantly enhance CPT-induced apoptosis in N2a NCs. Only a modest increase in apoptotic levels was observed compared to untreated controls (Supplementary Fig. 10B).

Thus, while both shRNA constructs efficiently reduced Cited2 expression, the magnitude of the apoptotic phenotype differed between them, suggesting that the contribution of Cited2 to CPT-induced cell death may be more limited or context-dependent.

Moreover, in contrast to the other experiments, in which CPT showed only a non-significant tendency to increase apoptosis under control conditions (Figs. 5A,B; 7B; Supplementary Fig. 7A,B), this effect was statistically significant in the current experiment (Supplementary Fig. 10B), highlighting CPT’s potential to induce apoptosis in the absence of E2F-related effects.

## Discussion

**Fig. 8:**
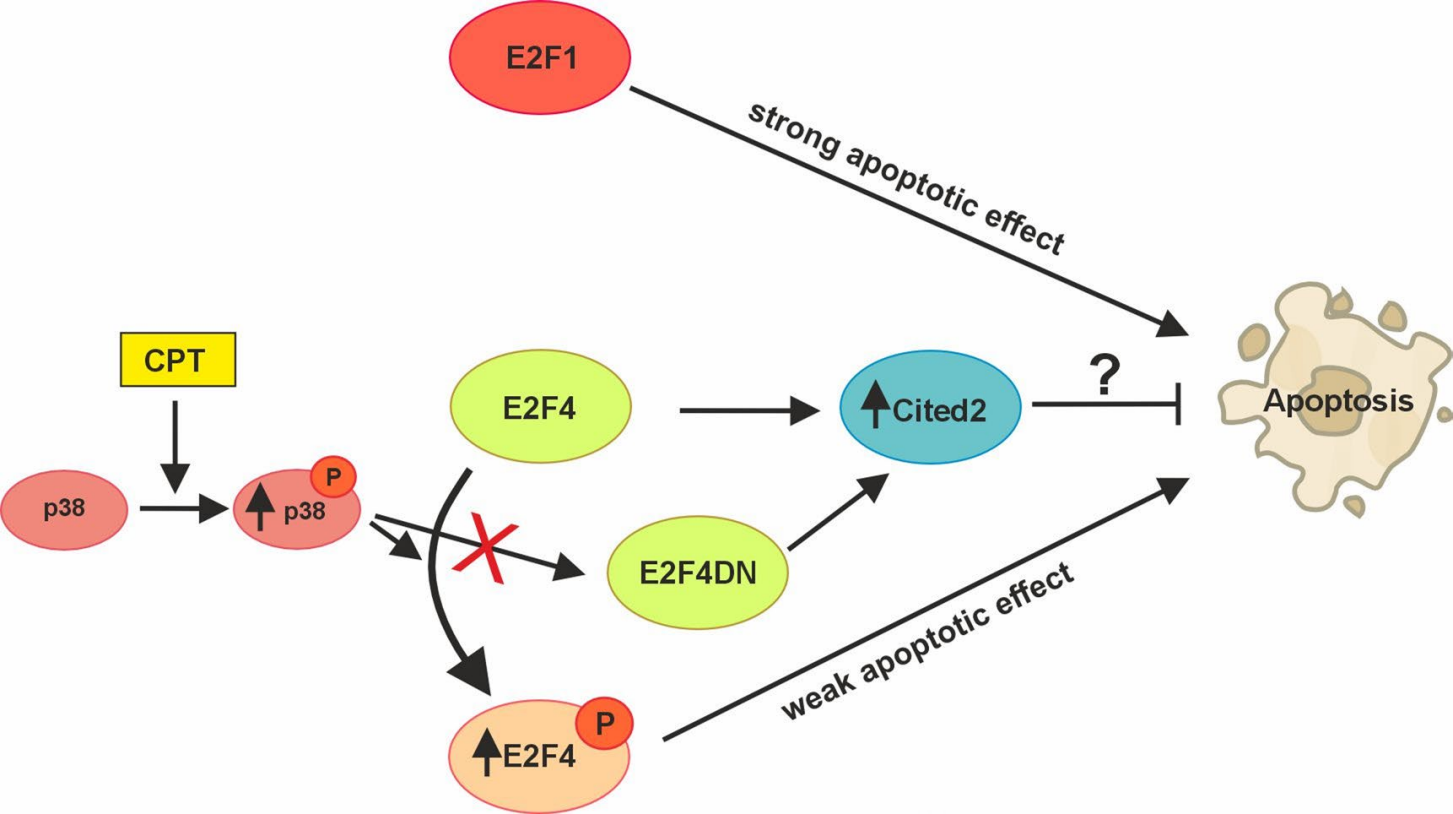
Scheme illustrating the proposed mechanism of E2F4DN to favor the survival of CPT-treated N2a NCs. Under normal conditions, E2F4 facilitates the expression of Cited2, which might trigger antiapoptotic signaling in N2a NCs. Genotoxic stress induced by CPT leads to p38^MAPK^ activation, which phosphorylates E2F4 at the Thr249/Thr251 motif. This facilitates a weak proapoptotic effect of E2F4 that mimics the effect of E2F1 as a strong proapoptotic trigger in CPT-treated cells. E2F4DN, which cannot be phosphorylated at the Thr249/Thr251 motif, maintains the capacity of E2F4 to induce Cited2 expression and, together with other putative mediators, its antiapoptotic effect.

In this study we have demonstrated that the transcription factor E2F4 can be detected in different phosphorylation states in N2a NCs, showing a spatial pattern that changes when the nucleus is compared to the cytosol. The phosphorylation pattern of exogenous E2F4 changes in response to CPT treatment, due to the activation of p38^MAPK^, a stress kinase that phosphorylates E2F4 in the cytosol within its Thr248/Thr250 motif. This phosphorylation, independently of the phosphorylation state of E2F4 in other residues, reduces the capacity of E2F4 to induce *Cited2* expression and likely acting through other unknown mechanisms, thus favoring CPT-dependent apoptosis. A phosphomimetic form of E2F4 containing the Thr248Glu/Thr250Glu mutation acts synergistically with CPT to induce apoptosis in N2a NCs, although with reduced potency compared to E2F1. In contrast, E2F4DN favors the survival of N2a NCs even under strong CPT-triggered genotoxic stress. This stresses the relevance of the phosphorylation of E2F4 by p38^MAPK^ at the Thr248/Thr250 motif in the apoptotic effect of E2F4 in N2a NCs subjected to genotoxic stress (Fig. 8).

We have shown that, in N2a NCs, E2F4 can be detected in different phosphorylation states. This confirms previous results in colorectal adenomas and intestinal epithelial cells^17^, in C33A and U937 cells^35^, as well as a study in mouse embryonic stem cells showing that E2F4 contains three threonines, one tyrosine, and six serines susceptible to become phosphorylated, in addition to Thr249 (26). Remarkably, the incubation of the cell extracts with AP resulted in a shift towards two high mobility bands, suggesting that two main forms of E2F4 are present in N2a NCs, likely differing from each other by a covalent modification. This contrasts with the results from (35), who found only one band in C33A cells treated with λ phosphatase. In N2a NCs, E2F4 could be covalently modified by acetylation of its K96 residue^8^, a modification that would theoretically result in a higher mobility when resolved in SDS-PAGE gels^59^. Alternatively, the cleavage of E2F4 in N2a NCs, as previously shown for E2F1^60^, could also explain the presence of this high-mobility band.

Our study revealed a different phosphorylation pattern of E2F4 when nuclear and cytosolic extracts were compared, with enhancement of low-mobility bands in the former subcellular compartment, whose particular identity changed depending on the E2F4 variant. Therefore, the translocation of E2F4 to the nucleus seems to be associated with its phosphorylation state, as previously demonstrated^17^, who shown that ERK1/2-mediated phosphorylation of E2F4 on Ser244 and Ser384 promotes its nuclear localization in proliferating intestinal cells.

CPT treatment resulted in the phosphorylation of the modified form of E2F4WT at Thr248, resulting in the increase of the intensity of band *d* in the cytosol. The conclusion that the phosphorylated residue of E2F4 that becomes phosphorylated in response to CPT is Thr248 was supported by the observation that the intensity of band *d* did not change upon CPT treatment in both E2F4DN- and E2F4CA-expressing N2a NCs. In addition, immunostaining of N2a NCs with an antibody specific for the phosphoThr249 epitope of endogenous E2F4 confirmed that CPT induces the phosphorylation of E2F4 at Thr249.

We provide evidence that the phosphorylation of E2F4 in response to CPT is triggered by p38^MAPK^, a finding consistent with previous studies demonstrating that CPT and Irinotecan, a CPT derivative that also inhibits topoisomerase I, can both induce p38^MAPK^ activity in cancer cells^61,62^, thus indicating that p38^MAPK^ can be specifically activated in response to DNA damage^63^. In this regard, we have confirmed that CPT treatment of N2a NCs results in time-dependent activation of p38^MAPK^ but not of JNK, a related stress kinase. The activation of p38^MAPK^ by CPT was paralleled by detection by immunocytochemistry of the phosphoThr249 epitope in N2a NCs, an effect that could be prevented with the p38^MAPK^-specific inhibitor SB203580 but not by JNK inhibition with SP600125. Interestingly, SB203580 is a known inhibitor of the isoforms p38α and p38β^64,65^, suggesting that these isoforms are those involved in the phosphorylation of E2F4 in response to cellular stress. These results are consistent with our finding that the domain of mouse E2F4DN where the conserved threonines Thr249 and Thr251 are located functionally interacts with p38α but not with JNK1, as evidenced by cell-free kinase assays. Our results also indicate that SB203580 is not able to reduce the phosphorylation levels of E2F4 at 0–4 h. A possible explanation for this observation is that under basal conditions E2F4 is phosphorylated by p38γ or p38δ, while a switch to the p38α/p38β isoforms may take place at 6–8 h. This view is consistent with the fact that the canonic sequence of human p38^MAPK^ employed to generate the mAb used in this study dramatically differs in the p38γ isoform. Therefore, this antibody could not detect the presence of p38γ in the cell extracts illustrated in Fig. 3A.

p38^MAPK^ activation can act as a switch between prosurvival and proapoptotic signaling depending on the strength of the DNA damage^66–68^. In this regard, highly concentrated CPT treatment represents a strong signal leading to p38^MAPK^-dependent apoptosis^61,68^. The effect of CPT is known to be potentiated by E2F1 in a cell cycle-independent manner^2,24,25^. In line with these observations, we found that E2F1-overexpressing N2a NCs underwent apoptosis 48 h after treatment with 10 μM CPT. E2F4 has been shown to regulate the proapoptotic effect of E2F1^26^, but the role of E2F4 phosphorylation in this paradigm was unknown. We have demonstrated in this study that the CPT-dependent apoptotic response can be potentiated by E2F4, although with reduced capacity compared to E2F1, depending on its Thr248 phosphorylation state. The expression of the Thr248/Thr250 phosphomimetic form of E2F4 (E2F4CA), significantly potentiated the CPT-induced apoptosis in N2a NCs. In contrast, E2F4DN, which lacks the capacity to become phosphorylated by p38^MAPK^ as it contains the Thr248Ala/Thr250Ala mutation, prevented this cell death.

Our results have revealed that the mechanism used by E2F4DN to prevent CPT-dependent cell death in N2a NCs does not rely in its capacity to induce the expression of DNA damage repair genes^8,69^. This lack of repair effect of E2F4DN is likely due to the strong DNA damage triggered by the high concentration of CPT that was used, which resulted in a reduction of nuclei showing punctate γH2AX labeling concomitant with an increase of nuclei with a diffuse γH2AX immunostaining. This diffuse pattern has previously been described in response to strong DNA damage such as that observed in response to UV irradiation^70^.

We have shown that the lack of apoptotic effect of E2F4DN in CPT-treated N2a NCs might depend on Cited2, a transcriptional regulator that can trigger either apoptosis of cell survival depending on the cell context^54,55^. This possibility rests on two distinct lines of evidence. On the one hand, E2F4DN, but not E2F4CA, upregulates *Cited2* expression before CPT-dependent p38^MAPK^ activation. On the other hand, *Cited2* knockdown might potentiate the apoptotic effect of CPT and prevented the antiapoptotic effect of E2F4DN. If Cited2 would be acting independently of E2F4, then the increase of apoptosis observed in N2a NCs transduced with shCITED2 in the presence of CPT would have been reduced by E2F4DN co-expression, but this was not the case (Fig. 7C). Same levels of apoptosis were observed in cells expressing shCITED2 and shCITED2/E2F4DN, a finding compatible with the possibility that Cited2 acts downstream of E2F4DN.

However, experiments using a second independent shRNA, despite achieving efficient Cited2 depletion, resulted in a more modest apoptotic phenotype. This variability suggests that the contribution of Cited2 to the antiapoptotic effect of E2F4DN may be partial and possibly influenced by knockdown dynamics, target sequence context, or compensatory mechanisms. Therefore, while our data are consistent with a model in which Cited2 acts downstream of E2F4DN to modulate CPT-induced apoptosis, they do not definitively establish a causal relationship. Additional complementary approaches, such as rescue experiments or genetic ablation strategies, will be required to fully define the role of Cited2 in this context. Moreover, given the pleiotropic transcriptional functions of E2F4, it remains possible that other apoptosis-related genes^4^ contribute in parallel or synergistically to the observed phenotype.

Interestingly, our results in N2a NCs differ from those described by a previous study that found an opposed activity of Cited2 in primary cortical neurons^26^. In this latter paradigm, CPT prevents the capacity of E2F4 to repress the capacity of E2F1 to induce the expression of Cited 2, followed by apoptosis.

In summary, we have uncovered in this study a putative mechanism of action of E2F4DN that may participate in its multifactorial therapeutic capacity as a homeostasis regulator (Fig. 8). This mechanism, which derives from its resistance to become phosphorylated by p38^MAPK^ under genotoxic stress, might be relevant for AD^8–10^, since the affected neurons in this disease have been shown to undergo genotoxic stress^71,72^. However, it is important to note that these findings were obtained using differentiated neuron-like cells derived from the mouse N2a neuroblastoma line. While this model is widely used for mechanistic studies, its tumor origin and species-specific differences may influence stress responses, signal transduction, and apoptotic pathways compared to human neurons^73^. Therefore, further validation in human neuronal models, such as iPSC-derived neurons or AD-specific cellular systems, will be necessary to fully assess the translational relevance of these results.

## Materials and methods

### Antibodies

Primary and secondary antibodies together with detailed information about their manufacturers and dilutions that were used for the different procedures in which they were used are shown in Supplementary Tables 1 and 2, respectively. The immunopurified rabbit polyclonal antibody recognizing the phosphoThr249 epitope of mouse E2F4 (p-E2F4) was previously described^9^.

### Peptides

A control peptide containing the transactivator of transcription (TAT) sequence of human immunodeficiency virus-1 (HIV-1) (TAT peptide: YGRKKRRQRRR), and a peptide containg the TAT sequence followed by the amino acids 237–256 of the mouse E2F4 sequence (NCBI accession number NP_683754) containing the Thr249Ala/Thr251Ala mutation (TAT-E2F4DN peptide: YGRKKRRQRRRQESSPPSSPQLTAPAPVLGS) were produced by Synpeptide Co. Ltd.

### Adenoviral vectors

The coding sequence of human E2F1 (NCBI accession number NM_005225), preceded by the HA tag, or of human E2F4 (NCBI accession number NM_001950), followed by the 6xHis taq, were synthesized by GenScript and cloned into the pcDNA3 plasmid (Invitrogen) to generate the pE2F1 and pE2F4 plasmids. Then, two mutations were introduced into the pE2F4 plasmid to generate the pE2F4DN (Thr248Ala/Thr250Ala) and pE2F4CA (Thr248Glu/Thr250Glu) plasmids. Finally, serotype 5 adenoviral vectors (Ad5) carrying the human E2F1, wild-type E2F4 (E2F4WT), E2F4DN, and E2F4CA coding sequences were generated as previously described^74^. To this end, recombinant plasmids were generated containing the human synapsin 1 (hSyn1) promoter, the coding sequence of each of these human proteins, and a mutant woodchuck hepatitis virus post-transcriptional regulatory element sequence (WPRE3SL)^9^, flanked by the adenovirus-specific inverted terminal repeats (ITRs) as well as all the genes necessary to generate the vector, except for E1, which codes for the E1A and E1B proteins. These latter proteins were constitutively expressed by the Q packaging cell line BI-HEK_293A, which was transfected with the respective recombinant plasmids described above. In addition, an Ad5 vector expressing a mouse *Cited2*-specific shRNA carrying EGFP as a reporter (Ad5-m-CITED2-shRNA; Cat. No: shADV-255523) was purchased to Vector Biolabs. A second Ad5 vector was generated by UPV (Autonomous University of Barcelona) to express a mouse Cited2-specific shRNA targeting base pairs 1232–1252 of the sequence (NCBI accession no. NM_001428637) and carrying EGFP as a reporter gene.

### Cell cultures

Mouse N2a cells^22^ were obtained from the American Type Culture Collection (ATCC) and maintained in Dulbecco’s modified Eagle medium (DMEM) (Gibco) supplemented with 10% FBS (Gibco), and 0.5% penicillin/streptomycin (P/S) (Gibco). A maximum of 15 passages were given to each cell batch to maintain the characteristics of the cells. For neuronal differentiation, N2a cells were plated at 20,000 cells/cm^2^ in DMEM supplemented with 1% FBS without antibiotics and cultured for 18–20 h before performing the experiments. For immunocytochemistry, cells were plated at the same conditions as above on coverslips (Menzel Glässer, 10 mm) pretreated with 65% nitric acid for 18–20 h, and then extensively washed with MilliQ water and sterilized with absolute ethanol.

### Adenoviral transduction

For transduction, N2a NCs were cultured for 48 h in the presence of 85 infectious units (IU)/cell of each Ad5 vector. For co-transduction experiments, 60 IU/cell were used for each Ad5 vector (120 IU in total). Around 75–95% of cells were efficiently transduced with these infectious titers. Biosecurity procedures have been approved by the Ethics Committee of CSIC.

### DNA damage induction

To induce severe DNA damage to N2a NCs, 10 µM CPT (Cell Signaling Technology), prepared in dimethyl sulfoxide (DMSO), was added to the cultures during different time points depending on the experimental design. CPT is a known topoisomerase I inhibitor^38^ that causes double-strand breaks in the replication forks that are associated with DNA replication as well as in the DNA supercoils that are formed during transcription^75^.

### p38^MAPK^ and c-Jun N-terminal kinase (JNK) inhibition

The p38^MAPK^ inhibitor SB203580 (Cell Signaling Technology) was used at 10 µM, and the JNK kinase inhibitor SP600125 (Calbiochem) was used at 25 µM. Both compounds are competitive inhibitors that bind to the ATP binding pocket of these kinases to prevent their catalytic activity^44,76^. Both inhibitors were prepared in DMSO, and added 1 h before CPT treatment. In all cases, control cultures were treated with an equivalent volume of DMSO. Total DMSO concentration was always below 0.5%.

### RNA extraction and cDNA synthesis

N2a NCs were washed with phosphate buffered saline (PBS) and total RNA was then extracted with Qiazol (Qiagen) following the instructions of the manufacturer. The concentration of RNA in each sample was then measured using NanoDrop One UV–Vis (ThermoFisher Scientific), and 5 µg of total RNA were used for the synthesis of the cDNA in a 20 µl reaction mix containing 4 µl of 5 × reaction buffer (Invitrogen), 40 units of RNAsin (Promega), 250 ng/µl of random primers (Invitrogen), 10 mM dNTPs (Biotools), 10 mM dithiothreitol (DTT) (Invitrogen), and 200 units of SuperScript IV (Invitrogen), following the instructions of the reverse transcriptase manufacturer.

### qRT-PCR

qRT-PCR was performed with the QuantStudio 3 Real-Time PCR system (Thermo Fisher Scientific) using SYBR Green (Cultek) in 15 µl reaction volume containing 1 µl cDNA and the upstream and downstream oligonucleotides (see Supplementary Table 3) at 10 µM.

### Cell extracts

Cultures were placed on ice, washed with ice-cold PBS, and incubated for 3 min with 200 μl of cold extraction buffer containing 20 mM Tris–HCl (Roche) pH 6.8, 1 mM EDTA (Merck), 1% Triton-X100 (Sigma), 0.5% sodium dodecyl sulfate (Sigma-Aldrich), 10 mM β-mercaptoethanol (Sigma-Aldrich), and 1 × cOmplete, Mini, EDTA-free, Protease Inhibitor Cocktail (Roche). 1 × Phosphatase Inhibitor Cocktail 2 (Sigma-Aldrich) was added to the cell lysing buffer in experiments where preservation of phosphoproteins was mandatory. Cell extracts were then scraped with a rubber policeman and homogenized using a 22G needle. Protein was then quantified using the Protein Assay Dye Reagent Concentrate system (BioRad).

### Subcellular fractionation

Subcellular fractionation was performed using a modification of a previously described protocol^77^. Briefly, cultures were placed on ice, washed with ice-cold PBS, and lysed with 500 μl of cold lysis buffer containing 10 mM HEPES (Sigma-Aldrich) pH 7.6, 10 mM NaCl (Merck), 1 mM KH_2_PO_4_ (Merck), 5 mM NaHCO_3_ (Merck), 5 mM EDTA (Merck), 1 × cOmplete, Mini, EDTA-free, Protease Inhibitor Cocktail. 1 × Phosphatase Inhibitor Cocktail 2 was added to the cell lysing buffer in experiments where preservation of phosphoproteins was mandatory. Cell lysates were then scraped with a rubber policeman and homogenized by 10 passages through a 22G needle, and an aliquot of the cell lysate was kept as a lysate control. Cell nuclei were spun down at 300 × g for 10 min and the nuclear pellet was kept at 4 ºC. The supernatant containing membranes and cytosol was then microfuged at 16,000 × g for 45 min at 4 °C. The supernatant (cytosol fraction) was removed and concentrated with Vivaspin 500 Centrifugal Concentrator filters (cutoff, 3 kDa) (Sartorius) as described by the manufacturer. In addition, lysis buffer containing 1% Triton X-100 was added to the pellet (membrane fraction). Finally, nuclear proteins were extracted for 15 min under frequent vortexing in high-salt buffer containing 5 mM HEPES pH 8.0, 420 mM NaCl, 0.25 mM MgCl_2_ (Merck), 0.1 mM EDTA, 12.5% glycerol (PanReac), 1 × cOmplete Mini, EDTA-free, protease inhibitor cocktail, and 1 × Phosphatase Inhibitor Cocktail 2. The extract was then centrifuged at 16,000 × g for 10 min at 4 °C and the supernatant (soluble nuclear fraction) was kept on ice.

### p38^MAPK^ and JNK assays

A luminiscent p38^MAPK^ assay was performed using the p38α Kinase Enzyme System (Promega) and the ADP-Glo kinase Assay kit (Promega) following the instructions of the manufacturer. The JNK1 Kinase Enzyme System (Promega) together with the ADP-Glo kinase Assay kit was used to evaluate the activity of JNK. TAT and TAT-E2F4DN peptides were dissolved at 10 mM in 1 × kinase reaction buffer [40 mM Tris–HCl pH 7.5, 20 mM MgCl_2_, 50 μM DTT and 0.4 μg/μl bovine serum albumin (Sigma-Aldrich)]. Phosphorylation reactions (5 μl final volume) were performed in Nunc 384-well white plates (ThermoFisher Scientific) containing 10 ng of p38α (or of JNK1), 200 ng of the p38α (or of JNK1) substrate provided by the manufacturer, and the TAT or TAT-E2F4DN peptides at the indicated concentrations. Kinase reactions were initiated by adding ATP at a final concentration of 30 μM. After 30 min at room temperature (RT), 5 μl of ADP-Glo reagent was added and incubated at RT for 40 min to stop the kinase reactions. Then 10 μl of Kinase Detection Reagent was added and incubated for 30 min at RT. The light generated was measured with a BMG FLUOstar OPTIMA Microplate reader (BMG Labtech).

### Dephosphorylation assay

A cell extract volume containing 16 µg of protein was incubated for 2 h at 37 ºC in 200 µl 1 × FastAP reaction buffer (ThermoFisher Scientific) containing 100 U FastAP thermosensitive alkaline phosphatase (AP) (ThermoFisher Scientific). Then, the reaction was stopped by adding 50 µl of 250 mM EDTA. Finally, reactions were concentrated with Vivaspin 500 Centrifugal Concentrator filters (cutoff, 3 kDa), as described by the manufacturer.

### Western blotting and image analysis

Cell extracts and subcellular fractions obtained as described above or AP-treated proteins were boiled for 5 min in 1 × Laemli buffer. The extracts were fractionated by SDS PAGE on acrylamide gels of the appropriate concentration or Tris–Glycine Precast Gels (4–15%) (NZYTECH) (PCNA), and transferred to 0.45 µm Immobilon-FL polyvinylidene difluoride (PVDF) transfer membranes (Millipore). The membranes were incubated for 1 h with Intercept (TBS) Blocking Buffer solution (LI-COR) (IBB), and then incubated overnight at 4 °C with the appropriate antibody in IBB. After washing the membranes three times in TBS containing 0.1% Tween 20 (TBS-T), they were incubated for 1 h at RT with a 1/15,000 dilution of secondary antibodies in IBB containing 0.1% Tween 20. Finally, they were washed again with TBS-T as described above, followed by a final wash with TBS. The protein bands were then visualized using the Odyssey CLx Infrared Imaging System (LI-COR) without overexposure or contrast enhancement. All images were acquired under identical exposure conditions for each fluorescence channel. Band intensities were quantified using LicorLite using the “asign shape” command for background subtraction. The intensity for each E2F4-specific band obtained in subcellular fractions was quantified using the command “plot profile” from Fiji (ImageJ2; https://imagej.net/software/fiji). The intensity of bands corresponding to proteins of interest were normalized to the intensity of actin or lamin B1 in the same lane and related to total amount of E2F4. Full uncropped gels and blots images are included as Supplementary Information.

### Immunocytochemistry

Immunocytochemistry was performed in N2a NCs cultured on glass coverslips fixed for 15 min at RT with 4% paraformaldehyde (PFA; Merck). After three washes with PBS, cells were permeabilized and blocked with PBS containing 0.1% Triton-X100 (PBS-T) supplemented with 10% FBS (blocking medium) for 1 h. Coverslips were then incubated for 2 h at RT with blocking medium and the appropriate primary antibody. After three washes in PBS-T, coverslips were incubated for 1 h in blocking medium containing the appropriate secondary antibody and 0.1 μg 4’,6-diamidine-2'-phenylindole (DAPI; Sigma-Aldrich). After three washes with PBS-T plus one additional washing step with PBS, coverslips were mounted with InmunoSelect Antifade Mounting Medium (Dianova) on glass slides. Images of the cells were taken by confocal microscopy.

### Direct ELISA

The peptides CSPQLT(phosphoT)PTP (pE2F4 peptide) and CSPQLTTPTP (E2F4 peptide) corresponding to mouse E2F4 protein (NCBI accession number NP_683754) were synthesized by Nanbiosis (https://www.nanbiosis.es) and conjugated to bovine serum albumin (BSA) (pE2F4-BSA and E2F4-BSA, respectively) using the Thermo Scientific Imject Maleimide-Activated BSA Spin Kit (Fisher Scientific; Cat. No. 11874035). The conjugated proteins were coated onto ELISA plates, and their interaction with the anti-pE2F4 antibody was detected using the Universal HRP-Conjugated Streptavidin ELISA Kit (antibodies.com; Cat. No. A326620).

### Confocal microscopy and image analysis

Confocal images were acquired with a Leica SP5 inverted confocal microscope. In all analyses, a range of 10–35 images of 1 µm thick were taken per stack at 40 × magnification so that the entire thickness of the cells present in the microscope field is included, and then the maximum projection was obtained. Images used for the analysis of γH2AX were taken at 40 × magnification with 2 × zoom.

Image analysis was performed using Fiji software. For the analysis of caspase-3 activation, around 3000 cells were analyzed per condition and experiment. The number of cells positive for caspase-3 cleaved at Asp175 were counted and quantified as the percentage of DAPI labeling. Pyknotic nuclei were identified based on characteristic features, including strong DAPI fluorescence intensity, nuclear condensation, and reduced nuclear size compared with normal nuclei. The percentage of pyknotic cells was calculated relative to the total number of DAPI-positive nuclei. For the analysis of DNA damage foci through γH2AX immunostaining, around 900 cells per condition and experiment were classified based on the nuclear labeling pattern as well as the number of foci present in their nuclei. For the analysis of the phosphoE2F4 vs. E2F4 ratio, DAPI was used as a reference for cell identification (300 cells per condition and experiment) and the same setting was used for both phosphoE2F4 and E2F4 labeling channels. Briefly, after adjusting the cell outlines using the “threshold” command, the “fill holes” and the “watershed” commands were used to improve the selection of the cells. Then, the region of interest (ROI) tool was used to measure the “integrated density” of each cell, which was subtracted from the average value of the background signal outside the cells. Fiji software was used for the analysis of the 6xHis immunofluorescence levels using the rectangle tool. The pixel intensity mean was quantified for each non-transfected cell to set up the background threshold. Cells were considered to be positive for the staining if they showed mean values above this threshold.

### Statistical analysis

Statistical analyses were carried out using Prism software (version 9) (https://www.graphpad.com/features; GraphPad). Quantitative data are represented as the mean ± standard error of the mean (SEM). Statistical tests used in this study and the number of independent samples for each experimental group are specified in the figure legends. Differences were considered to be statistically significant with a *p*-value < 0.05.

## Supplementary Information

Below is the link to the electronic supplementary material.

Supplementary Material 1

Supplementary Material 2

## Data Availability

All data generated or analyzed during this study are included in this published article and its supplementary information files. Original, unprocessed versions of the full-length gels and blots have been included in the Supplementary Information.doc file. Material are available from the corresponding author on reasonable request.
