# Supplementary material for: Resistance of E2F4DN to p38MAPK phosphorylation reduces genotoxic cell death in N2a neuron-like cells
Source: Sci Rep. 2026 May 30;16:24860. doi: 10.1038/s41598-026-54592-z (PMC13457616; doi:10.1038/s41598-026-54592-z)
Supplement: Supplementary file 1 [file 41598_2026_54592_MOESM1_ESM.docx]

**Resistance of E2F4DN to p38^MAPK^ phosphorylation reduces genotoxic cell death in NCs-like cells**

**Aina M. Llabrés-Mas, Noelia López-Sánchez, Alberto Garrido-García, Vanesa Cano-Daganzo, Irene Camacho-Olmos & José María Frade**

**Supplementary Tables**

**Supplementary Table 1.** Primary antibodies

| Protein | Manufacturer | Reference | Host | Application | Dilution |
| --- | --- | --- | --- | --- | --- |
| E2F4 | Millipore | MABE160 | Mouse | ICQ  WB | 1:200  1:2000 |
| P-E2F4 |  |  | Rabbit | ICQ | 1:50 |
| p-p38 | Cell Signaling | 4511 | Rabbit | WB | 1:1000 |
| p38 | Cell Signaling | 8690 | Rabbit | WB | 1:2000 |
| Cleaved Caspase-3 (Asp175) | Cell Signaling | 9661 | Rabbit | ICQ | 1:150 |
| 6xHis tag | Abcam | ab18184 | Mouse | WB | 1:1000 |
| Lamin B1 | Abcam | ab133741 | Rabbit | WB | 1:4000 |
| Actin | Millipore | MAB1501R | Mouse | WB | 1:10000 |
| p-JNK | Cell Signaling | 9252 | Rabbit | WB | 1:1000 |
| JNK | Cell Signaling | 9251S | Rabbit | WB | 1:1000 |
| EGFP | Nacalai Tesque | 04404-84 | Rat | ICQ | 1:500 |
| γ H2AX | Abcam | ab26350 | Mouse | ICQ | 1:300 |
| MAP2 | Abcam | ab5392 | Chicken | ICQ | 1:250 |
| Synapsin 1 | Abcam | ab64581 | Rabbit | ICQ | 1:400 |
| PCNA | Abcam | ab2426 | Rabbit | WB | 1:1000 |
| GAPDH | Invitrogen | AM4300 | Mouse | WB | 1:10000 |
| Na+/K+ ATPase α1 | Merck | 06-520 | Rabbit | WB | 1:2000 |

**Supplementary Table 2.** Secondary antibodies

| Antibody | Host | Conjugated with | Manufacturer | Reference | Aplication | Dilution |
| --- | --- | --- | --- | --- | --- | --- |
| Anti-mouse | Donkey | Alexa 647 | Invitrogen (Life Technologies) | A31571 | ICQ | 1/1000 |
| Anti-mouse | Goat | Alexa 594 | Invitrogen (Life Technologies) | A11032 | ICQ | 1/1000 |
| Anti-mouse | Goat | Alexa 568 | Invitrogen (Life Technologies) | A21043 | ICQ | 1/1000 |
| Anti-mouse | Goat | IRDye 680RD | Li-cor | 926-68070 | WB | 1/7500 |
| Anti-rabbit | Goat | Alexa 568 | Invitrogen (Life Technologies) | A11036 | ICQ | 1/1000 |
| Anti-rabbit | Donkey | Alexa 488 | Invitrogen (Life Technologies) | A21206 | ICQ | 1/1000 |
| Anti-rabbit | Goat | IRDye 800CW | Li-cor | 926-32211 | WB | 1/7500 |
| Anti-rat | Goat | Alexa 647 | Invitrogen (Life Technologies) | A21247 | ICQ | 1/1000 |

**Supplementary Table 3.** Oligonucleotides used in qPCR

| Transcript | Species | Position (bp) | NCBI accesión # | Size (bp) | Sequence 5’ –> 3’ |
| --- | --- | --- | --- | --- | --- |
| *Rps18* | *M. musculus* | 120-139 | NM_011296 | 73 | **U:** AATAGCCTTCGCCATCACTG |
|  |  | 173-192 |  |  | **D:** GTCTGCTTTCCTCAACACCA |
| *Cited2* | *M. musculus* | 878-897 | NM_010828 | 76 | **U:** CGCCCAATGTCATAGACACT |
|  |  | 932-953 |  |  | **D:** CGGTCCAAACCCATTTCTATCA |
| *Syn1* | *M. musculus* | 2108-2129 | NM_013680.4 | 103 | **U:** AGCTCAACAAATCCCAGTCTCT |
|  |  | 2191-2210 |  |  | **D:** CGGATGGTCTCAGCTTTCAC |
| *E2F4* | *H. sapiens* | 1291-1310 | NM_001950 | 138 | **U:** TGATGTGCCTGTTCTCAACC |
|  | Woodchuck hepatitis virus | 1166-1186 | NC_004107 |  | **D:** GCATTAAAGCAGCGTATCCAC |

**Supplementary Fig. 1** Differentiation of N2a neuroblastoma cells into N2a NCs. **A** Representative bright field images of N2a neuroblastoma cells cultured with DMEM supplemented with FBS 10% (10 % FBS) or FBS 1% (1% FBS). Box: high magnification to visualize the neurites (arrows) that can be observed in N2a neuroblastoma cells cultured with 1% FBS (i.e. N2a NCs). Bar: 25 μm. **B** Representative immunostaining of N2a neuroblastoma cells cultured with 10% FBS or 1% FBS, using antibodies for the neuronal markers MAP2 (MAP2) and Synapsin 1 (Syn1). Cell nuclei were labeled with DAPI (blue). Arrow: MAP2-positive process. White labeling was obtained using secondary antibodies conjugated to Alexa Fluor 647. Bar: 25 μm. **C** Quantification of *Syn1* mRNA by real-time quantitative PCR (RT-qPCR) normalized to the *Rps18* housekeeping gene in cDNA obtained from N2a NCs cultured with either FBS 10% or FBS 1%. RT: reverse transcriptase. *p<0.05 (Two-way ANOVA, followed by Tukey's HSD post-hoc test).

**Supplementary Fig. 2** Method for quantifying 6×His fluorescence intensity in Ad5.E2F4WT-6xHis-, Ad5.E2F4DN-6xHis-, and Ad5.E2F4CA-6xHis-transduced cells. **A** Representative image of unspecific 6xHis immunostaining in non-transduced cells, showing a crescent-shaped pattern. White rectangle oriented perpendicular to the crescent-like immunolabeling: area selected to analyze green fluorescence per cell. Bar: 25 μm. **B** Representative histogram showing the distribution of pixel intensities within the rectangular region of a single control cell. This analysis allows quantification of the mean fluorescence intensity per cell. Most pixels display very low intensity values. **C** Quantification of the mean fluorescence intensity per cell in 71 randomly selected control cells. Average mean intensity per cell obtained: 15.6622 (identified in panels B and E with a green arrow). Transduced cells are considered positive for the 6xHis tag when their mean fluorescence value is above this value. **D** Representative image showing 6xHis-specific immunostaining of Ad5.E2F4WT-6xHis-transduced cells. White rectangle: area selected to analyze green fluorescence per cell. Bar: 25 μm. **E** Representative histogram showing the distribution of pixel intensities within the rectangular region of a single histogram of an Ad5.E2F4WT-6xHis-transduced cell.

**Supplementary Fig. 3** Cellular fractionation markers in N2a NCs. Representative western blots of whole cell extracts (homogenates) and the indicated subcellular fractions from N2a NCs, using antibodies against GAPDH, Na^+^/K^+^ ATPase α1, and Actin are shown. Notice that GAPDH can be detected in the cytosolic fraction as a band of ~36 kDa. PCNA is specific for the nuclear fraction, and a 112-kDa band specific for Na^+^/K^+^ ATPase α1 only appears in the membrane fraction (non-specific bands were found in the nuclear fraction). Actin was used as a loading marker. While actin is best known for its cytoskeletal functions, multiple studies have demonstrated the presence of distinct actin pools associated with the plasma membrane and the nucleus. These pools contribute, respectively, to membrane organization and mechanics^S1^ and to transcriptional regulation and chromatin dynamics^S2^. Note that the bands shown were obtained from different gels. Specifically: GAPDH, box 22 in Supplementary Material Figure 1; PCNA, box 23 in Supplementary Material Figure 1; Na^+^/K^+^ ATPase α1, box 24 in Supplementary Material Figure 1; Actin, box 25 in Supplementary Material Figure 1.

**Supplementary Fig. 4** Specificity of anti-pE2F4 assessed by direct ELISA. A phospho-Thr249 E2F4 peptide and an unphosphorylated control peptide encompassing Thr249 were conjugated to bovine serum albumin (pE2F4-BSA and E2F4-BSA, respectively), adsorbed onto ELISA plates, and incubated with the indicated dilutions of a P-E2F4–specific antibody. Absorbance values were plotted as a function of antibody dilution. R, Pearson correlation coefficient.

**Supplementary Fig. 5** Increase of E2F4 protein in CPT-treated N2a NCs. **A** Representative western blot of cell extracts from N2a NCs at the indicated time points after 10 µM CPT treatment, using an anti-E2F4-specific antibody. Lamin B1 (Lamin) was used as a loading marker. **B** Regression analysis of the E2F4 protein levels at the indicated time points after 10 µM CPT treatment. R^2^=0.9646; p=0.0354. Note that the bands shown were grouped from cropped blots obtained from different gels. Specifically: E2F4, box 26 in Supplementary Material Figure 1; Lamin, box 27 in Supplementary Material Figure 1.

**Supplementary Fig. 6** Regulation of apoptosis by E2F1 in CPT-treated N2a NCs. Percentage of active caspase-3-positive N2a NCs related to total DAPI-positive nuclei at the indicated time points. ***p<0.005 (Two-way ANOVA, followed by Tukey's HSD post-hoc test). Bar: 25 μm.

**Supplementary Fig. 7** Regulation of apoptosis by E2F4 phosphorylation in CPT-treated N2a NCs. Percentage of pyknotic N2a NCs transduced with the indicated forms of E2F4 and treated with 10 µM CPT for 24 h (**A**) or 48 h (**B**) n: number of independent experiments. **p<0.01 (Two-way ANOVA, followed by Tukey's HSD post-hoc test).

**Supplementary Fig. 8** Cited2 mRNA levels, normalized to the Rps18 housekeeping gene, in cDNA obtained from N2a NCs non-transduced (N.T.) or transduced with either EGFP (EGFP), E2F4DN-6xHis (E2F4DN) or E2F4CA-6xHis (E2F4CA) at time 0 h (Fig. 7A). *p<0.05 (One-way ANOVA).

**Supplementary Fig. 9** Regulation of *Cited2* expression by a *Cited2*-specific shRNA in N2a NCs. **A** Left panel: representative immunostaining of N2a NCs transduced with an Ad5 vector co-expressing a *Cited2*-specific shRNA together with EGFP (shCITED2), using antibodies against EGFP. Cell nuclei were labeled with DAPI (blue). Right panel: quantification of EGFP-positive N2a NCs related to DAPI-positive nuclei. White labeling was obtained using secondary antibodies conjugated to Alexa Fluor 647. Bar: 25 μm. **B** Quantification of *Cited2* mRNA by RT-qPCR normalized to the *Rps18* housekeeping gene in cDNA obtained from N2a NCs transduced with either EGFP or an Ad5 vector co-expressing a *Cited2*-specific shRNA together with EGFP. **p<0.01 (unpaired Student’s *t* test).

**Supplementary Fig. 10** Regulation of Cited2 expression by an Ad5 vector co-expressing EGFP and another Cited2-specific shRNA (shCITE2-2), and participation of Cited2 in the CPT-induced apoptotic cell death in N2a NCs. **A** Quantification of *Cited2* mRNA by RT-qPCR normalized to the *Rps18* housekeeping gene in cDNA obtained from N2a NCs transduced with shCITED-2. **p<0.01 (unpaired Student’s *t* test). **B** Percentage of active caspase-3-positive N2a NCs transduced with shCITED2-2, and cultured for 32 h with 10 µM CPT, related to total DAPI-positive nuclei. n: number of independent experiments. *p<0.05; ***p<0.001 (Two-way ANOVA, followed by Tukey's HSD post-hoc test).

**Supplementary References**

S1. Köster, D. V. & Mayor, S. Cortical actin and the plasma membrane: inextricably intertwined. *Curr. Opin. Cell Biol.* **38**, 81-89 (2016). doi: 10.1016/j.ceb.2016.02.021

S2. Serebryannyy, L. & de Lanerolle, P. Nuclear actin: The new normal. *Mutat. Res.* **821**, 111714 (2020) doi: 10.1016/j.mrfmmm.2020.111714
