# Supplementary material for: Resistance of E2F4DN to p38MAPK phosphorylation reduces genotoxic cell death in N2a neuron-like cells
Source: Sci Rep. 2026 May 30;16:24860. doi: 10.1038/s41598-026-54592-z (PMC13457616; doi:10.1038/s41598-026-54592-z)
Supplement: Supplementary file 2 [file 41598_2026_54592_MOESM2_ESM.docx]

8

9

2

1

3

4

7

6

5

10

12

11

**C**

14

13

18 22

17 22

16 22

15 22

**D**

GAPDH

PCNA

23 22

22 22

Na^+^/K^+^

ATPase

α1

Actin

24 22

25 22

**E**

27 22

26 22

**F**

28 22

**Supplementary Material Fig. 1.** Uncropped WBs used to construct Figs. 1, 2, 3 and Supplementary Figs. 3 and 5. **A** WBs used to create Fig. 1C (upper) and Fig. 1D (bottom). **B** WBs used to create Fig. 2A. Two rounds of primary antibody incubation were performed to identify 6xHis and Actin. **C** WBs used to create Fig. 3A (upper) and Fig. 3C (middle and bottom). **D** WBs used to create Supplementary Fig. 3. **E** WBs used to create Supplementary Fig. 5. Boxes identify the regions that were used to create the Figures. The molecular weight markers identified in panel **F** can be extrapolated to the rest of panels as the same markers were used for all the blots. Red numbers identify the cropped bands shown in the main and supplementary figures. These numbers are indicated in the respective figure legends.
